# Calculating the Number of Cluster Heads Based on the Rate-Distortion Function in Wireless Sensor Networks

**DOI:** 10.1155/2014/602875

**Published:** 2014-07-01

**Authors:** Mingxin Yang, Jingsha He, Yuqiang Zhang

**Affiliations:** ^1^College of Computer Science and Technology, Beijing University of Technology, Beijing 100124, China; ^2^College of Economics Management, Hebei University of Science and Technology, Shijiazhuang 050018, China; ^3^School of Software Engineering, Beijing University of Technology, Beijing 100124, China

## Abstract

Due to limited resources in wireless sensor nodes, energy efficiency is considered as one of the primary constraints in the design of the topology of wireless sensor networks (WSNs). Since data that are collected by wireless sensor nodes exhibit the characteristics of temporal association, data fusion has also become a very important means of reducing network traffic as well as eliminating data redundancy as far as data transmission is concerned. Another reason for data fusion is that, in many applications, only some of the data that are collected can meet the requirements of the sink node. In this paper, we propose a method to calculate the number of cluster heads or data aggregators during data fusion based on the rate-distortion function. In our discussion, we will first establish an energy consumption model and then describe a method for calculating the number of cluster heads from the point of view of reducing energy consumption. We will also show through theoretical analysis and experimentation that the network topology design based on the rate-distortion function is indeed more energy-efficient.

## 1. Introduction

Wireless sensor networks (WSNs) have become more and more widely used in a variety of applications. In most large-scale WSNs, individual wireless sensor nodes will first transmit sensed data to cluster heads which will then forward the data to the sink node. Due to limited resources in the sensor nodes, power consumption has become a primary consideration during data transmission. In general, it would consume more energy for data transmission than for data processing. Therefore, reducing the amount of data transmission is a very important means of reducing the total amount of energy consumption in WSNs. Among possible approaches for reducing data transmission, hierarchical network topology based data fusion has been considered as an effective means of reducing communication traffic. With data fusion, a certain level of distortion may have to be tolerated by actual applications, which makes it unnecessary to transmit all the collected data to the sink and the amount of data that should be transmitted to the clusters and to the sink depends on the level of distortion that can be tolerated. Hierarchical data fusion topology, which can be developed based on the rate-distortion function, can help to reduce communication traffic while making sure that the amount of data collected and transmitted is sufficient to meet the requirement of real applications.

Castanedo summarized the state of the data fusion research and described many relevant studies [1]. Hall and Llinas introduced the emerging technology of multisensor data fusion [2]. Huang et al. proposed a novel weight-based clustering decision fusion algorithm (W-CDFA) to detect target signal in wireless sensor networks [3]. Abdulsalam and Ali introduced a new data aggregation algorithm for uniform, nonuniform, and evolving networks while maintaining data accuracy [4]. Ahvar et al. proposed an energy-aware routing protocol (ERP) for query-based applications in WSNs, which offers the tradeoff between traditional energy balancing and energy saving objectives and supports soft real-time packet delivery [5]. Yang et al. proposed a method for achieving an optimal number of aggregation points with a power consumption model and analyzed the effect of different numbers of aggregation points on the performance [6]. But the work did not consider the issue of distortion. Without measurement on data compression, there may be too much or too little information to the sink node. A large amount of data will lead to redundant information while causing an unnecessarily high level of energy consumption. Although a small amount of data can reduce energy consumption, the sink node may not be able to restore the original message, making the data less useful for the sink node. Akyildiz et al. introduced the concept of wireless sensor networks that have been made viable by the convergence of microelectromechanical system technologies, wireless communications, and digital electronics [7]. Deng and Huang established a communication model and analyzed energy consumption under two different circumstances, that is, collecting data once per round and collecting data several times per round [8] in which an optimal data collection scheme is designed by determining the optimal times of data collection to optimize data acquisition for hierarchical networks. The work also analyzed the differences among data acquisition schemes by assuming that all the sensor nodes have the same initial energy condition in WSNs. However, this paper did not study the exact corresponding number of aggregators. Heinzelman et al. studied the application of the networks in harsh network environment with severe resource constraints and proposed application-specific protocol architecture in contrast to the traditional layered approaches [9]. Yang et al. proposed a more reasonable energy consumption model, that is, the optimal energy consumption model (OECM) [10]. Yu et al. proposed a method to design an optimal path to acquire data in sparse wireless sensor networks based on a multiplicatively weighted Voronoi diagram [11].

In this paper, we propose a method for the calculation of the number of cluster heads based on the rate-distortion function after establishing an energy consumption model according to the data fusion framework in WSNs. Our energy consumption model includes three parts: data transmission from wireless sensor nodes to the cluster heads, data compression or aggregation in the cluster heads, and data transmission from the cluster heads to the sink. We will evaluate our proposed method on energy consumption based on the above established energy consumption model and the rate-distortion function to demonstrate the energy efficiency of the method.

The remainder of this paper is organized as follows. In Section 2, we introduce some preliminary knowledge required for our discussion which includes the theoretical derivation on the computability of the number of cluster heads, the concept of distortion, and the rate-distortion function. In Section 3, we introduce an energy consumption model and propose a method for calculating the number of cluster heads based on the rate-distortion function. In Section 4, we show that the design of the network topology based on the rate-distortion function is more energy-efficient than that without considering the distortion. In Section 5, we describe some experiment that we have performed and present and analyze the simulation results. Finally, in Section 6, we conclude this paper in which we also discuss some future research directions.

## 2. The Preliminaries

### 2.1. Computability of the Number of Cluster Heads

In WSNs, hierarchical topology for data fusion is generally preferred in which each round of the collection process will result in a fixed number *K* of sensor nodes as the cluster heads. At the beginning of each round of the process, every sensor node generates a random number between 0 and 1 and compares the random number with a probability value *P*_*i*_(*t*). If the random number is smaller than *P*_*i*_(*t*), the sensor node will periodically broadcast an ADV message to its neighboring nodes to inform that it will be the cluster head. The formula for the probability value *P*_*i*_(*t*) [12] is defined as follows:

(1) $$\begin{array}{c} P_{i}\left(t\right)=\{\begin{array}{cc} \frac{K}{N-K*\left(r\mathrm{mod}\left(N/K\right)\right)} & C_{i}\left(t\right)=1\\ 0 & C_{i}\left(t\right)=0, \end{array} \end{array}$$

where *P*_*i*_(*t*) is the probability that node *i* would act as the cluster head at time *t*. Let *N* denote the number of sensor nodes in a WSN, *K* denote the number of cluster heads at each round, and *r* denote the current working round. *C*_*i*_(*t*) would indicate whether node *i* has the right to become a cluster head at time *t*. When *C*_*i*_(*t*) = 1, node *i* is entitled to become a cluster head at time *t* and when *C*_*i*_(*t*) = 0 node *i* is not entitled to become a cluster head at time *t*.

It is clear that each node will be able to function as a cluster head once within *N*/*K* rounds. Every node has the opportunity to serve as the cluster head; those nodes that have already served as the cluster heads in the first round can no longer serve as the cluster heads in the next *N*/*K* − 1 rounds. Those nodes that can serve as the cluster head fall off; the probability that a remaining node can become a cluster head would go up. After *N*/*K* − 2 rounds, the probability that the remaining nodes who never serve as cluster head can become a cluster head would be 1.

**Lemma 1 .**

For a WSN with *N* nodes, if there are *K* clusters upon completing each round, then *P* = *K*/*N* and every node can become a cluster head once during *N*/*K* rounds.

**Proof**

In round (*r* + 1), if the probability that a remaining node can become a cluster head at time *t* is *P*_*i*_(*t*), the expectation of the cluster head denoted as *N*_ch_ is as follows:

(2) $$\begin{array}{c} E\left(N_{\text{ch}}\right)=\overset{N}{\underset{i}{\sum }}P_{i}\left(t\right)*1=K. \end{array}$$

Since the number of nodes that have not served as the cluster heads in the previous *r* rounds is *N* − *K*∗*r*, after *N*/*K* rounds, every node should become a cluster head exactly once. Regarding the meaning of *C*_*i*_(*t*), symbol ∑_*i*=1_^*N*^ *C*_*i*_(*t*) represents the total number of nodes that can serve as the cluster heads at time *t* and we can then get the following formula:

(3) $$\begin{array}{c} E\left[\overset{N}{\underset{i=1}{\sum }}C_{i}\left(t\right)\right]=N-K*\left(r\mathrm{mod}\frac{N}{K}\right). \end{array}$$

According to Formulas (2) and (3), we can get the mathematical expectation for the cluster head number, which is *E*(*N*_ch_):

(4) E(Nch)=E(∑iNPi(t)∗Ci(t))=∑iNPi(t)∗E(∑iNCi(t))=(N−K∗(rmod⁡NK)) ×KN−K∗(rmod⁡(N/K))=K,

where *N*_ch_ is the number of cluster heads, *E*(*N*_ch_) is the expectation of cluster head number, *P*_*i*_(*t*) is the probability that node *i* will act as a cluster head at time *t*, and *C*_*i*_(*t*) indicates whether node *i* has the right to function as a cluster head at time *t*. Again, *N* is the number of sensor nodes in a WSN, *K* is the number of cluster heads after each round, and *r* is the current working round.

### 2.2. The Rate-Distortion Function

Generally, it is not necessary to transmit every piece of the collected data to the sink. As a result, a certain level of information distortion may occur, which must be under the tolerance level of the sink. For a given source entropy *H*(*X*) and allowed distortion, the amount of information from the source should be as small as possible, which is derived as the theoretical value from the information rate-distortion function.

#### 2.2.1. The Function

Let us define the discrete information source as follows [13]:

(5) $$\begin{array}{c} \left[\begin{array}{c} X\\ P \end{array}\right]=\left[\begin{array}{cccc} x_{1} & x_{2} & \cdots & x_{n}\\ p\left(x_{1}\right) & p\left(x_{2}\right) & \cdots & p\left(x_{n}\right) \end{array}\right]. \end{array}$$

The output sequence after transmission through a channel is $y=\left[\begin{array}{cccc} y_{1} & y_{2} & \cdots & y_{m} \end{array}\right]$. The distortion function is a nonnegative function which is a quantitative description of the receiver *y*_*j*_ from source *x*_*i*_. Then, let us arrange all the *d*(*x*_*i*_, *y*_*j*_), where *i* = 1,2,…, *n* and *j* = 1,2,…, *m*, and the resulting matrix [*d*] can be expressed as follows:

(6) $$\begin{array}{c} \left[d\right]=\left[\begin{array}{cccc} d\left(x_{1},y_{1}\right) & d\left(x_{1},y_{2}\right) & \cdots & d\left(x_{1},y_{m}\right)\\ d\left(x_{2},y_{1}\right) & d\left(x_{2},y_{2}\right) & \cdots & d\left(x_{2},y_{m}\right)\\ \vdots & \vdots & \vdots & \vdots \\ d\left(x_{n},y_{1}\right) & d\left(x_{n},y_{2}\right) & \cdots & d\left(x_{n},y_{m}\right) \end{array}\right]. \end{array}$$

This matrix [*d*] is called the distortion matrix.

In the matrix, the nonnegative function *d*(*x*_*i*_, *y*_*j*_) can be selected to meet specific needs, such as the squares cost function, the absolute cost function, and the uniform cost function.

#### 2.2.2. Distortion Measurement Flow

The distortion function matrix is also called the distortion matrix [*D*], where the upper limit of the distortion can be calculated based on the distortion matrix.

**Figure 1:**
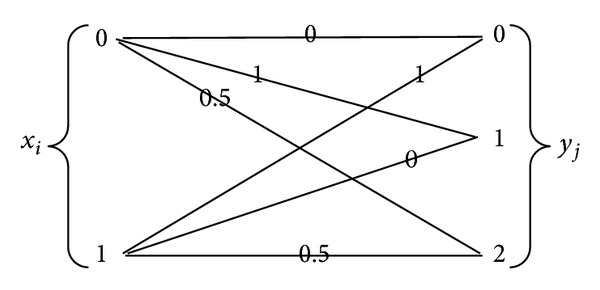
The distortion measurement flow.

Let us suppose that *X* ∈ {0,1}, *Y* ∈ {0,1, 2}, *d*(0,0) = *d*(1,1) = 0, *d*(0,1) = *d*(1,0) = 1, *d*(0,2) = *d*(1,2) = 0.5. The distortion measurement flow can then be described in Figure 1.

The corresponding distortion matrix is then $D=\left[\begin{array}{ccc} 0 & 1 & 0.5\\ 1 & 0 & 0.5 \end{array}\right]$.

#### 2.2.3. Information Rate-Distortion Function

Suppose the rate of information transmission through a channel with capacity *C* is *R*. If *R* > *C*, information at the source should be compressed or aggregated so that the compressed transmission rate *R*′ is lower than the channel capacity *C*. Let us assume that the predetermined average distortion is *D* and the average distortion of the compressed source is $\bar{D}$; for a given source, we should make the amount of the information transmitted as small as possible. All the channels that can satisfy the criterion $\bar{D}\leq D$ are called the permitted channels *B*_*D*_.

We can therefore find a channel *p*(*Y*/*X*) among the permitted *B*_*D*_ channels so that the channel transmission rate *I*(*X*, *Y*) is minimized for a given source to push information through this channel. All the channels that can meet the above condition are called the rate-distortion function, namely,

(7) $$\begin{array}{c} R\left(D\right)=\underset{p(Y/X)\in B_{D}}{\min}I\left(X,Y\right). \end{array}$$

For a discrete memoryless source, the rate-distortion function can then be expressed as follows [13]:

(8) R(D)=min⁡p(yj/xi)∈BD∑i=1n ∑j=1mp(xi)p(yjxi)·log⁡p(yj/xi)p(yj),

where *p*(*x*_*i*_)  (*i* = 1, 2, …, *n*) is the probability distribution at the source, *p*(*y*_*j*_)  (*j* = 1, 2, …, *m*) is the probability distribution at the receiver, and *p*(*y*_*j*_/*x*_*i*_)  (*i* = 1, 2, …, *n*; *j* = 1, 2, …, *m*) is the transition probability distribution.

#### 2.2.4. The Rate-Distortion Function for Different Types of Sources

An information source is a source for generating information or information sequence. Actually, there may be many information sources. The output of these sources is the information of a single symbol. Therefore, the number of such symbols is limited and countable. We hence use *x*, which is a one-dimensional discrete random variable, to describe the output of the information source, which is called a discrete source. When the output of the source is a continuous function, which means that the value of the source is both continuous and random, the information source is called a continuous information source. A discrete source would include a probability source, such as a binary source and an *n*-element source. A continuous source would include a Gaussian source and so on. There is an upper bound on the degree of distortion and the rate-distortion function of several sources among which *D* is the distortion, *σ* ^2^ is the mean square error, *α* is the value of the distortion function, and *R*(*D*) is the rate-distortion function as described below.

(1) For a binary source:
  (9) R(D)=H(p)−H(Dα)=−pln⁡p−(1−p)ln⁡⁡(1−p)+Dαln⁡Dα+(1−Dα)ln⁡(1−Dα),
  where $\left[\begin{array}{c} X\\ p(x_{i}) \end{array}\right]=\left[\begin{array}{cc} x_{1} & x_{2}\\ p & 1-p \end{array}\right]p\leq 1/2$, and
  (10) $$\begin{array}{c} \left[D\right]=\left[\begin{array}{cc} 0 & \alpha \\ \alpha & 0 \end{array}\right]\alpha >0. \end{array}$$
(2) For two-element equal probability source:
  (11) $$\begin{array}{c} D_{\max}=\frac{1}{2}\alpha \end{array}$$
  (12) $$\begin{array}{c} R\left(D\right)=\ln2-H\left(\frac{D}{\alpha }\right), \end{array}$$
  where *p* = 1/2 and $[D]=\left[\begin{array}{cc} 0 & \alpha \\ \alpha & 0 \end{array}\right]\alpha >0$.
(3) For an *n*-element equal probability source:
  (13) $$\begin{array}{c} D_{\max}=\left(1-\frac{1}{n}\right)\alpha \end{array}$$
  (14) $$\begin{array}{c} R\left(D\right)=\ln n+\frac{D}{\alpha }\ln\frac{D/\alpha }{n-1}+\left(1-\frac{D}{\alpha }\right)\ln\left(1-\frac{D}{\alpha }\right), \end{array}$$
  where *p*(*x*_*i*_) = 1/*n*, (*i* = 1 ~ *n*) and $d(x_{i},y_{j})=\{\begin{array}{cc} 0 & i=j\\ \alpha & i\neq j \end{array}$.
(4) For a one-dimensional Gaussian source:
  (15) $$\begin{array}{c} R\left(D\right)=\{\begin{array}{cc} \frac{1}{2}\log_{2}\frac{\sigma^{2}}{D} & D<\sigma^{2}\\ 0 & D\geq \sigma^{2}. \end{array} \end{array}$$

## 3. Calculating the Number of Cluster Heads of Data Fusion

### 3.1. Number of Cluster Heads Based on the Rate-distortion Function

Assuming that all wireless sensor nodes are distributed in a circular area with radius “*a*”, and the sink node is located in the center of the circle, and that there are one or more clusters in the same circular area. The process of transmitting data from regular sensor nodes to the sink node is to that of transmitting the data to the corresponding cluster heads, and then aggregating on the sink node along the way. Thus, the transmission paths form a hierarchical network. Assuming also that the center of the circular area that is covered by cluster *C* is denoted by a node (*x*_0_, *y*_0_) and the distance that the sensor nodes in cluster *C* can transmit data to the sink node is *S*.

In the following formula, assuming that *a* is the radius of the circular area *C*, *α*′ is the energy consumption coefficient, *α*_1_ is the loop energy consumption coefficient, *α*_2_ is the antenna energy consumption coefficient, *n* is the number of wireless sensor nodes in the circular area, *r* is the rate of data transmission, *δ* is the routing influence coefficient, *k* is the number of cluster heads in the circular area, *d*_char_ is the regional characteristic radius, *γ* is the compression ratio, *c* is the number of over-compression, and *β* is data compression coefficient, then, if one circle with radius a consists of *k* number of circular clusters and the radius of each of the clusters is *x*, we can get the following formula:

(16) $$\begin{array}{c} k\pi x^{2}=\pi a^{2} \end{array}$$

(17) $$\begin{array}{c} x=\frac{a}{\sqrt{k}}. \end{array}$$

We can further get the formula for distance *S* as follows:

(18) S=n×δπa2×∬(x,y∈C)(x−x0)2+(y−y0)2dx dy=n×δπa2×∫02πdθ∫0a/kr2×r dr=n×δπa2∫02πdθ×13×r3/0a/k=n×δπa2×13×a3k3/2×θ/02π=2anδ3k3/2.

The amount of energy consumed by a network consists of three parts: *P*_1_, which is for the wireless sensor nodes in each circular area to transmit data to the cluster heads, *P*_2_, which is for the cluster heads to receive the data, and *P*_3_, which is for the cluster heads to transmit the data to the sink node. The formula for *P*_1_ in terms of *k* fusion nodes can be expressed as follows:

(19) $$\begin{array}{c} P_{1}=\frac{2\alpha^{\prime }anr\delta }{3k^{1/2}}. \end{array}$$

With an acceptable distortion *D*, the minimum amount of data *R*(*D*) is the amount of data transmitted and received by cluster heads. If *β* is the energy coefficient of data fusion at the cluster heads, the energy consumption for classical fusion is proportional to the amount of compressed data; that is,

(20) $$\begin{array}{c} P_{2}=\beta R\left(D\right). \end{array}$$

If the density of cluster heads is *k*/*πa* ^2^ in a circular area with *k* cluster heads, with the assumption of a linear compression model for the cluster heads, the following formula would hold:

(21) $$\begin{array}{c} P_{3}=\left(\gamma R\left(D\right)+c\right)\times \frac{2k\alpha^{\prime }a}{3}. \end{array}$$

We could then calculate energy consumption *E* based on Formulas (19), (20), and (21) as follows:

(22) $$\begin{array}{c} E=P_{1}+P_{2}+P_{3}. \end{array}$$

(23) $$\begin{array}{c} E=\frac{2\alpha^{\prime }anr\delta }{3k^{1/2}}+\beta R\left(D\right)+\left(\gamma R\left(D\right)+c\right)\times \frac{2k\alpha^{\prime }a}{3}. \end{array}$$

Since our purpose is to calculate *k* in order to minimize *E*, we can force *E* = 0 and the process is as follows:

(24) E=2α′anrδ3k1/2+βR(D)+(γR(D)+c)×2kα′a3−12×2α′anrδ3k3/2+(γR(D)+c)×2α′a3=0α′anrδ3k3/2=2α′a(γR(D)+c)3nrδk3/2=2(γR(D)+c)k3/2=nrδ2(γR(D)+c)k=(nrδ2(γR(D)+c))2/3,

where there exists *α*′ = (*α*_1_ + *α*_2_ *d*_char_)/*d*_char_.

### 3.2. Examples Using the Rate-Distortion Function

(1) When there exist two element probability sources such that $\left[\begin{array}{c} x\\ p(x) \end{array}\right]=\left\{\begin{array}{cc} 0 & 1\\ 1/2 & 1/2 \end{array}\right\}$ and $D=\left\{\begin{array}{cc} \alpha & 0\\ 0 & \alpha \end{array}\right\}$, we get the following:
  (25) Dmax⁡=12×α+12×0=α2,  Dmin⁡=0.R(D)=ln⁡2−H(Dα)=ln⁡2−[Dαln⁡Dα+(1−Dα)ln⁡⁡(1−Dα)].

When *D*_max⁡_ = *α*/2, *R*(*D*_max⁡_) = 2ln⁡2 and *k* = (*nrδ*/2(2*γ*ln⁡2 + *c*))^2/3^.

When *D*_min⁡_ = 0, *R*(*D*_min⁡_) = *R*(0) = *R*(*D*)_max⁡_ = 1 and *k* = (*nrδ*/2*c*)^2/3^.

When *R*(*D*)_min⁡_ = 0, *k* = (*nrδ*/2*c*)^2/3^.

(2) When there exists a one-dimensional Gauss source that meets the mean square error distortion criterion $R(D)=\{\begin{array}{cc} \left(1/2\right)\text{lo}{\text{g}}_{2}\left(\sigma^{2}/D\right) & D<\sigma^{2}\\ 0 & D\geq \sigma^{2} \end{array}$, we get the following:

When *D* = 0, *R*(*D*) → *∞*.

When *D* = *σ* ^2^, *R*(*D*) = 0.

## 4. A Model for Energy Consumption Based on the Rate-Distortion Function

### 4.1. The Network Energy Consumption Model

We reference the energy model in the LEACH protocol in our study, which consists of two phases: cluster establishment phase and stable data transmission phase. Regarding the different types of energy consumption, we assume that there are electron energy consumption, energy consumption of the power amplifier when a node transmits data, and electron energy consumption which can occur only when a node receives data in a WSN. If *S*_elec_ is the energy consumption for transmitting or receiving one bit of data, *lS*_elec_ is then the energy consumption for transmitting or receiving an *l*-bit message. Our power amplifier consumption adopts the free space model (FS) and the multipath fading model (MP) according to the distance between the sources and the sink node. When the distance *d* between two nodes is shorter than a threshold value, the FS model is applied. When the distance *d* between two nodes is longer than a threshold value but shorter than the maximum communication distance *d*′, the MP model is applied. Therefore, energy consumption *S*_*s*_ of a node's sending an *l*-bit message is as follows:

(26) $$\begin{array}{c} S_{s}=\{\begin{array}{cc} lS_{\text{elec}}+l\varepsilon_{fs}d^{2} & \;d<d_{0}\\ lS_{\text{elec}}+l\varepsilon_{mp}d_{\text{to-BS}}^{4} & d_{0}\leq d<d^{\prime }. \end{array} \end{array}$$

Assuming that there are *n* nodes and *k* clusters in a WSN, the distance from a node in the circular area to the base station is *d*_to-BS_, where *d*_0_ ≤ *d*_to-BS_ < *d*′, and the distance from the same node in the circular area to the cluster head is *d*_to-clus_, where 0 < *d*_to-clus_ < *d*_0_; if energy consumption during the establishment phase is *S*_0_, then *S*_0_ = (*k*/*n*)*S*_1_ + (1 − (*k*/*n*))*S*_2_, where *S*_1_ is the energy consumption of the cluster head and *S*_2_ is the energy consumption of the nodes within the cluster during the cluster establishment phase.

During the stable data transmission phase, if the energy consumption of a cluster head for receiving the information from the nodes within the cluster is *S*_re_, then

(27) $$\begin{array}{c} S_{\text{re}}=lS_{\text{elec}}. \end{array}$$

Similarly, if the energy consumption for data transmission from the cluster head to the base station is *S*_*f*-BS_, then

(28) $$\begin{array}{c} S_{f\text{-BS}}=lS_{\text{elec}}+l\varepsilon_{mp}d_{\text{to-BS}}^{4}. \end{array}$$

If the energy consumption for data transmission from sensor nodes to the cluster head is *S*_*f*-clus_, then

(29) $$\begin{array}{c} S_{f\text{-clus}}=ls_{\text{elec}}+l\varepsilon_{fs}d_{\text{to-clus}}^{2}. \end{array}$$

The whole WSN will die when the first node exits the network due to the depletion of energy. Therefore, we consider the amount of energy of the node that first dies as the amount energy of the whole network.

### 4.2. Proof of the Validity of the Model

We now show that the topological structure of data fusion based on the rate-distortion function is more energy-efficient than those not allowing any distortion in WSNs.

The energy consumption of the entire network consists of three parts: energy consumption for cluster establishment, energy consumption of the cluster heads for receiving data, and energy consumption of the cluster head for sending the data to the sink. Therefore, the following formula holds:

(30) Ei=rn+q[(nk−1)Sre+nkSag+Sf-BS]+(rn−q)Sf-clus,

where *E*_*i*_ is the initial energy of the node that dies first, *r*_*n*_ is the total number of rounds, *q* is the number of elected cluster heads, *S*_ag_ is the energy consumption of a cluster head for compressing one message, *S*_*f*-BS_ is the energy consumption for transmitting data from a cluster head to the base station, and *S*_*f*-clus_ is the energy consumption for transmitting data from a node to the cluster head. According to formula (22), the following formula holds:

(31) Ei=rn+q[(n(nrδ/2(γR(D)+c))2/3−1)Sre+n(nrδ/2(γR(D)+c))2/3Sag+Sf-BS]+(rn−q)Sf-clus=rn+q[(n1/3×[2(γR(D)+c)]2/3(rδ)2/3−1)Sre+n1/3×[2(γR(D)+c)]2/3(rδ)2/3Sag+Sf-BS]+(rn−q)Sf-clus.

From the above formula, the total amount of energy consumption of the whole network is proportional to *R*(*D*). If there exist *D* ∈ [0, *D*_max⁡_] and *R*(*D*)_max⁡_ = *R*(*D*_min⁡_) = *R*(0), *R*(*D*) would get the maximum value when *D* = 0. That is, when there is no distortion requirement, *E*_*i*_ attains its maximum value. Therefore, a WSN design based on the rate-distortion function is better than a one without considering distortion for the purpose of saving energy.

## 5. Experiment and Simulation Results

The purpose of our experiment, which was performed using Matlab, is to evaluate our proposed method by comparing it with the method proposed by Yang et al. [6] in terms of the total amount of network energy consumption. The simulation parameters are set as follows: *n* = 10000, *a* = 1000 m,  *α*_1_ = 5 × 10^−8^ J/b, *α*_2_ = 1 × 10^−10^ J/b, *d*_char_ = 22.36, *β* = 5 × 10^−8^ J/b, *γ* = 30%, *c* = 32 bit, *δ* = 0.95, and *r* = 160 b/sec.

**Figure 2:**
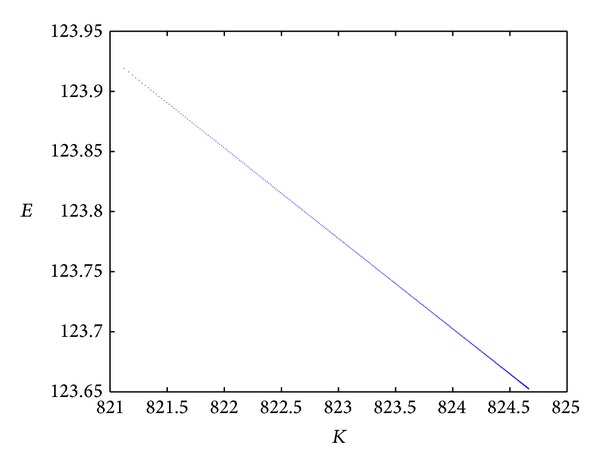
The total amount of network energy consumption of two-element source based on the rate-distortion function.

**Figure 3:**
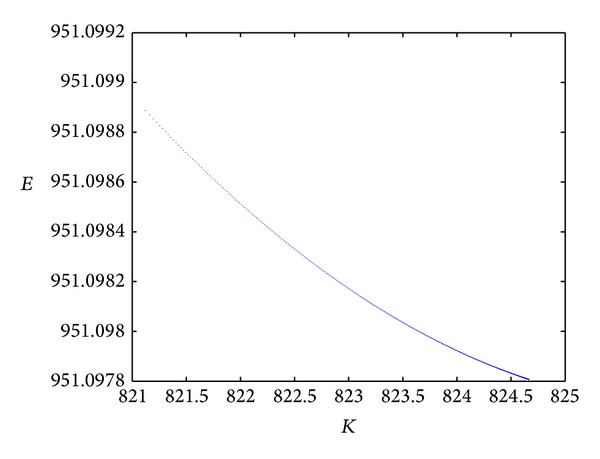
The total amount of network energy consumption without considering the rate-distortion function.

From Figures 2 and 3, we can see that the amount of energy that is consumed decreases along with the increase of *K*; that is, the energy consumption decreases along with an increase in the number of fusion nodes or cluster nodes. For the same number *K* in the range [821, 825], the total amount of network energy consumption of in the case of the two-element source based on the rate-distortion function shown in Figure 2 is lower than that shown in Figure 3 which does not employ the rate-distortion function. The experiment result is in line with the conclusion of the last section. Moreover, under the same simulation environment, the best result occurs when the number of fusion nodes is 821 for the Gaussian source. In a word, the method proposed in this paper is very suitable for the two-element source from the viewpoint of network energy consumption.

## 6. Conclusion

In this paper, we proposed a method for calculating the number of cluster heads based on the rate-distortion function. According to different requirements on information distortion and an established energy consumption model, the exact number of the cluster heads can be calculated for the purpose of data fusion. We showed that the proposed method is more effective through the means of mathematical proof. We also performed some analysis on the simulation results by using Matlab to demonstrate that the energy consumption of the model based on the rate-distortion function would consume less energy than the one that does not consider the factor of information distortion. In the future, we will perform the experiment and analysis based on some real network data to further improve the efficiency as well as energy consumption of our model for data fusion in WSNs.
